# Exploring the Vast Choice of Question Prompt Lists Available to Health Consumers via Google: Environmental Scan

**DOI:** 10.2196/17002

**Published:** 2020-05-29

**Authors:** Marguerite Clare Tracy, Heather L Shepherd, Pinika Patel, Lyndal Jane Trevena

**Affiliations:** 1 Ask, Share, Know: Rapid Evidence for General Practice Decisions Centre for Research Excellence School of Public Health The University of Sydney NSW Australia; 2 Centre for Medical Psychology and Evidence-based Decision-making (CeMPED) The University of Sydney NSW Australia

**Keywords:** question prompt list, shared decision making, environmental scan

## Abstract

**Background:**

There is increasing interest in shared decision making (SDM) in Australia. Question prompt lists (QPLs) support question asking by patients, a key part of SDM. QPLs have been studied in a variety of settings, and increasingly the internet provides a source of suggested questions for patients. Environmental scans have been shown to be useful in assessing the availability and quality of online SDM tools.

**Objective:**

This study aimed to assess the number and readability of QPLs available to users via Google.com.au.

**Methods:**

Our environmental scan used search terms derived from literature and reputable websites to search for QPLs available via Google.com.au. Following removal of duplicates from the 4000 URLs and 22 reputable sites, inclusion and exclusion criteria were applied to create a list of unique QPLs. A sample of 20 QPLs was further assessed for list length, proxy measures of quality such as a date of review, and evidence of doctor endorsement. Readability of the sample QPL instructions and QPLs themselves was assessed using Flesch Reading Ease and Flesch-Kincaid Grade Level scores.

**Results:**

Our environmental scan identified 173 unique QPLs available to users. Lists ranged in length from 1 question to >200 questions. Of our sample, 50% (10/20) had a listed date of creation or update, and 60% (12/20) had evidence of authorship or source. Flesch-Kincaid Grade Level scores for instructions were higher than for the QPLs (grades 10.3 and 7.7, respectively). There was over a 1 grade difference between QPLs from reputable sites compared with other sites (grades 4.2 and 5.4, respectively).

**Conclusions:**

People seeking questions to ask their doctor using Google.com.au encounter a vast number of question lists that they can use to prepare for consultations with their doctors. Markers of the quality or usefulness of various types of online QPLs, either surrogate or direct, have not yet been established, which makes it difficult to assess the value of the abundance of lists. Doctor endorsement of question asking has previously been shown to be an important factor in the effectiveness of QPLs, but information regarding this is not readily available online. Whether these diverse QPLs are endorsed by medical practitioners warrants further investigation.

## Introduction

The role of shared decision making (SDM) as a part of patient-centered care in clinical consultations is being increasingly recognized as having positive outcomes for patients [1]. Internationally, there are efforts to make SDM a part of routine health care [2]. The National Safety and Quality Health Service Standards (Second Edition) developed by the Australian Commission on Safety and Quality in Health Care include a statement that “Integral to the process is encouraging patients to be more involved and ask their doctor more questions during consultations” [3]. To facilitate patient question asking as a part of SDM, question prompt list (QPL) tools have been developed, and some have been evaluated and published in the peer-reviewed literature, some of which are available via the internet [4,5].

Increasingly, people are turning to the internet for health information [6]. Search engines are the predominant tool used by people to search for health information online [7]. In 2018, 78% of Australian adults reported using the internet to find health-related information [8]. Online QPLs are being used, and those using online question lists have been shown to prefer questions that specifically support SDM [9]. Physician endorsement has been found to be key to the successful implementation of QPLs into practice [4]. Similarly, a dominant factor for patient online health information seeking on the doctor-patient relationship is the doctor’s willingness to discuss the information [10].

With an increasing focus on question asking in consultations, many websites include lists of questions for someone to take to their doctor as an additional resource [11-13]. While there has been some research into the implementation of QPLs into practice, there has not been an assessment of the prevalence of such lists available online [5]. ​Given that online material can be created and hosted by anyone and there is no regulation of the quality of information or available tools, a study of these QPLs is warranted [14]. Assessing the readability of QPL resources as well as their prevalence is important in addressing usefulness.

Adequate literacy and health literacy of patients are important in the use of tools to support question asking and SDM [15]. In their systematic review published in 2012, Sørensen et al [16] identified several competencies of health literacy around having the ability to access, understand, and use information to make decisions about health. Supporting people with lower health literacy by making such tools readable and accessible is one way to increase access to SDM [15]. Several standards exist for the readability of patient information and other SDM tools such as decision aids, to increase the accessibility of materials for target audiences [17].

While standards exist for assessing the quality of patient information resources, such as the adaption of DISCERN for internet information [18,19], there is ongoing difficulty in assessing the quality of online medical information and resources as well as how users perceive and use that information [20-23]. In addition, there are factors other than the information itself that influence information preferences, such as domain bias [24] and webpage design [21]. For example, the extension “.com” is used for commercial sites, while “.edu,” “.gov,” and others denote non-commercial or government sites. Domain extensions have some bearing on how users view the information provided and their trust in the source [24,25].

Environmental scan processes have previously been used to assess available online decision aids and risk calculators [26,27]. They allow a real-time snapshot of the availability of online resources available to users. The aim of this study was to conduct an environmental scan to describe and assess the number and readability of QPLs readily available to health consumers in the online environment. 

## Methods

### Overview

We used a previously published methodology to conduct an environmental scan to search Google.com.au for question lists relating to patient-doctor clinical interactions [27]. Google.com.au was chosen as it is the most frequently used search engine in Australia (94.11% as of Dec 2018) [28]. To assess the number of question lists available to health care consumers online, 2 stages were needed. ​A third stage involved the assessment of the readability of a sample of QPLs and their instructions.

### Stage 1: Choice of Reputable Websites and Search Term Development

The authors have expertise and backgrounds in nursing and medicine; knowledge of organizations and websites used by health professionals and consumers was used to identify a range of reputable organizations’ websites to reflect the clinical areas in which QPLs have been studied [4]. The aim was to ensure that selected sites included both disease-specific lists (eg, cancer websites, parent information about their child’s attention deficit hyperactivity disorder) as well as sites with more generic lists, such as those with consumer health information. We also aimed to ensure a mix of local (Australian) and international organizations with patient-focused information. The final list of 22 URLs for these organizations was decided by consensus between the authors (Table 1). These websites were accessed via the URLs to confirm that they referred to, or included, QPLs.

Using previous systematic reviews of QPLs and citation snowballing, we found 11 terms in the published literature that have been used to describe patient question lists [4,29]. In addition, the reputable organization list websites were accessed to find the language and terms used to describe QPLs on these sites; there were a further 9 terms found. Using these 2 sources, we had a total of 20 search terms for use in Stage 2 of the scan (Table 2).

**Table 1:** URLs of the 22 selected reputable websites.

Site or organization type, organization		URL
**Consumer-directed organizations**		
	Healthdirect Australia Ltd.	healthdirect.gov.au
	Consumers Health Forum	chf.org.au
**Information about medicine or prescribing**		
	National Prescribing Service – Choosing Wisely	choosingwisely.org.au/home
	National Prescribing Service	nps.org.au
**Government entities**		
	Department of Health	health.gov.au
	Health Canada	canada.ca/en/health-canada
	NHS^a^ United Kingdom	nhs.uk
	Therapeutic Goods Administration	tga.gov.au
	NHS Networks (Ask 3 questions)	personcentredcare.health.org.uk/resources/ask-3-questions-materials
	Institute for Healthcare Improvement (Ask Me 3)	ihi.org/resources/Pages/Tools/Ask-Me-3-Good-Questions-for-Your-Good-Health.aspx
**Disease-specific organizations**		
	Cancer Council Australia	cancer.org.au
	Cancer Australia	canceraustralia.gov.au
	Raising Children Network	raisingchildren.net.au
	ADHD^b^ Australia	adhdaustralia.org.au
	Breast Cancer Network Australia	bcna.org.au
	Family Planning NSW^c^	fpnsw.org.au
**Pediatric organizations**		
	Royal Children’s Hospital (RCH) Melbourne	rch.org.au
	Sydney Children’s Hospital Network	schn.health.nsw.gov.au
	HealthyChildren.org (American Academy of Pediatrics)	healthychildren.org
**Quality and safety organizations**		
	Australian Commission for Safety and Quality in Health Care	safetyandquality.gov.au
	Agency for Healthcare Research and Quality	ahrq.gov
	Wiser Healthcare	wiserhealthcare.org.au

^a^NHS: National Health Service.

^b^ADHD: attention deficit hyperactivity disorder.

^c^NSW: New South Wales.

**Table 2:** Search terms derived from reputable sites and literature.

Search term	Source
Ask health professional questions	Wiser Healthcare
Ask for information	NPS^a^ MedicineWise
Questions to ask your doctor	Choosing Wisely, Cancer Council, Healthdirect
Questions to ask	Family Planning NSW^b^
Asking questions	Choosing wisely
Ask your health professional	NPS MedicineWise
Patient ask questions	Scottish Health Council (Ask Me 3 search)
Patients ask provider	Ask Me 3
Asking (these 3) questions during appointment	Ask 3 questions
Question prompt list	Literature
Question prompt sheet	Literature
Patient question prompt list	Literature
Patient question prompt sheet	Literature
Question sheet	Literature
Question list	Literature
Patient question aid	Literature
Shared decision-making tool	Literature
Patient agenda form	Literature
Patient agenda list	Literature
Patient question asking support tool	Literature

^a^NPS: National Prescribing Service.

^b^NSW: New South Wales.

### Stage 2: Search Strategy 

A systematic search of Google.com.au using each of the 20 search terms was then conducted by 2 independent researchers (MT and PP), one term at a time after clearing the browser cache. The first 100 URL results for each term searched were downloaded to Excel spreadsheets and included in the first round. Users of Google have a strong bias to the order of results presented by Google [30], and few users look for a result beyond the first results page [31]. There are only 10 results displayed on the results page with Google’s default settings. We aimed to assess the breadth of lists available with our search terms, which was the reason for assessing the first 100 results for each term.

#### Inclusion Criteria

Websites, accessed via search URLs, included in the evaluation needed to meet the following criteria: provide a list of questions; question lists were described as for use by patients, carers, or parents in medical consultations (general practitioner or specialist medical consultations); lists were freely accessible (without registration or requiring payment); lists were written in English; and lists were visible as part of the website, not requiring downloading to be viewed, such as video files.

#### Exclusion Criteria

Websites or lists were excluded if the question list stated it was for doctor’s use; the use of the question list required additional supporting software, such as third-party document viewers; the website required registration or incurred a cost to access the question list; the list was on a sponsored site, such as a paid site that appears before the search results; the list required downloading to be able to be viewed, such as video files; the list was provided in an academic paper, unless the journal was aimed at consumers and the list was visible; and questions list was not focused on general practice and specialist consultations (eg, counselling for entry into clinical trials). URLs blocked by the university security software system were also not included as they were deemed a potential threat​.

The inclusion and exclusion criteria were then applied to the data by opening each of the URL links, again by MT and PP. Discrepancies regarding inclusion were resolved using a third reviewer (HS). Where there were duplications of lists, such as where the question list was identified on the website as sourced from another site or the webpage linked to a list already included in the review, these sites were then excluded to ascertain the final number of unique lists.

Further data about the URLs, websites, and lists were also collected. All URLs were assessed for the URL domain extension (ie, .edu, .com, .org). A sample of 20 lists was collated to consist of 10 URLs from the reputable organizations and, for comparison, a further 10 lists from other sites were selected using random number generation and matching to URLs from the Excel spreadsheet. The sample lists were assessed for the date of creation, review, or update, if available (copyright date for the website was not considered to be an indication that the website material had been reviewed); any evidence of review of the page, such as the name of a writer or reviewer; and an assessment of the number of questions in the lists.

### Stage 3: Readability

To assess the readability of the QPLs and their instructions for use, we utilized the Flesch Reading Ease (FRE) and Flesch-Kincaid Grade Level (FKGL) scores. The FRE is one measure of the complexity of a piece of text and is a score between 0-100 calculated using average sentence length and the average number of syllables of words in the text [32]. The higher the FRE score, the easier the text is to read. The FKGL uses the same data with different weightings with a result that equates to the number of years of schooling (in the United States) required to read the text [33]. We applied these formulae to the sample lists. Each of the 20 URLs were accessed again, and both the online instructions for use (or the available preamble to the question list) and then the list of question(s) were copied into separate documents. The readability tool was then applied to the copied texts to calculate FKGL and FRE scores (Table 3).

**Table 3:** Description of reading ease scores, reproduced from Flesch [32].

Flesch Readability Scores (reading ease), points	Description of style
90-100	Very easy
80-89	Easy
70-79	Fairly easy
60-69	Standard
50-59	Fairly difficult
30-49	Difficult
0-29	Very confusing

## Results

**Figure 1:**
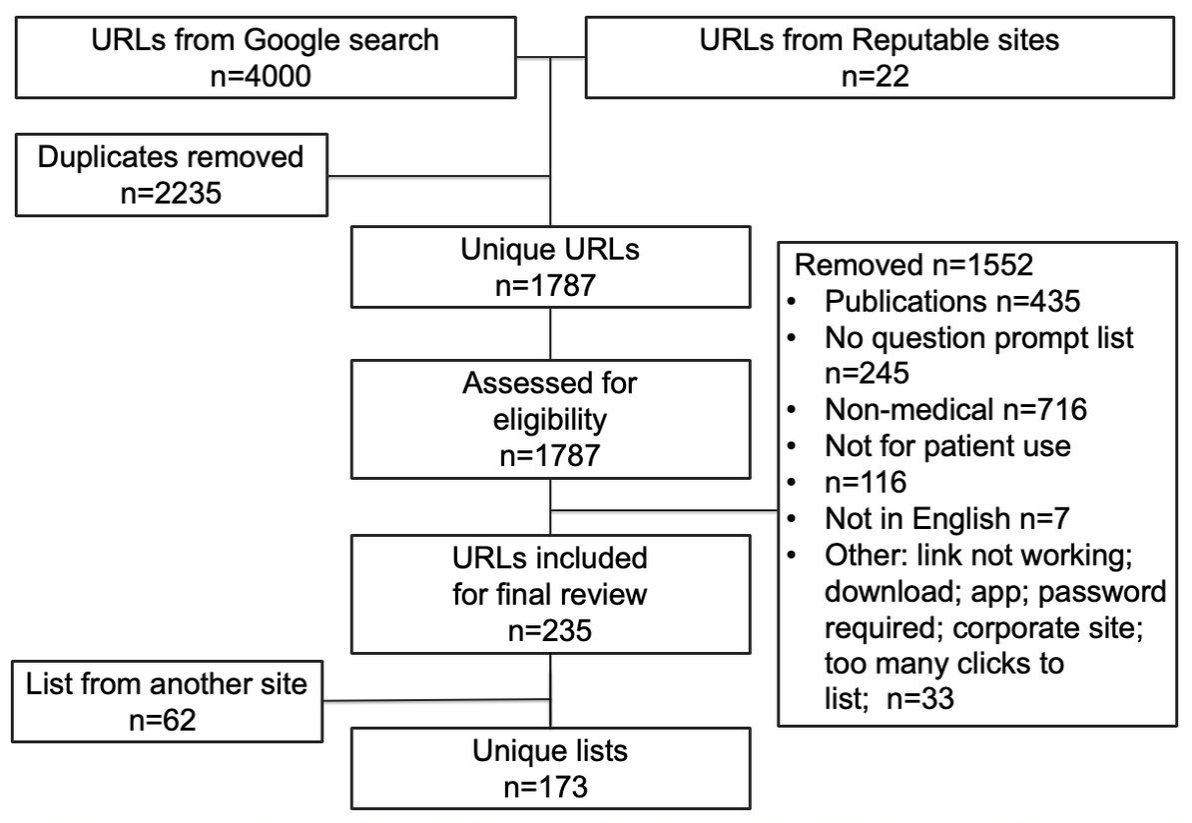
Search strategy and results.

Following removal of duplicates from the 4000 URLs and 22 reputable sites and the application of the inclusion and exclusion criteria, there were a total of 235 lists. A further review of websites revealed 62 instances of list duplication (eg, links to reputable lists and multiple uses of a list within an organization). Using our search method, 173 unique lists were identified. See Figure 1 for the study diagram of the search. There were 15 websites that had lists used, referred to, or had links to them from other websites; 9 of these were from our reputable website list, which accounted for 46 links.

We noted a wide range in the number of questions in resources, from a single question to over 200 questions in a single resource. The most common URL domain extension was .org (115/235, 48.9%), followed by .com (63/235, 26.8%), .gov (24/235, 10.2%), .edu (11/235, 4.7%), and country code extensions such as .uk and .ca (18/235, 7.7%). More detailed analysis of the sample of 20 lists found that the number of questions ranged from 3 to 56 (mean 20.5 questions, mode 3 questions, median 18 questions; Table 4). Half (10/20, 50%) of the sample lists had a date of creation or review, with the range being 2002 to the date of the preliminary review, October 29, 2018. Evidence of authorship of the QPL was available for 80% of the reputable sample sites and 40% of the other sample sites.

Table 4 also shows the readability data scores for the 20 sample lists and their online instructions. The FRE scores were higher for lists from the reputable sites compared to other sites and for question lists compared to instructions. Similarly, the grade level required to read question lists was lower than for the instructions, with reputable sites also requiring lower grade levels for readability of instructions than for other sites.

The readability was found to be easier on the reputable websites compared to the other websites, with scores showing content ranged from “very easy” to “fairly easy” for question lists and from “easy” to “difficult” for the instructions.

**Table 4:** Sample list data: readability of instructions and question lists, number of questions, source/author information, and date of creation or review.

Organization or website		Instructions and/or introduction for list				Question list				Number of questions		Evidence of source or author		Date of creation or review	
		Flesch readability		FKGL^a^		Flesch readability		FKGL							
**Reputable sites**															
	Wiser Healthcare		N/A^b^		n/a		85.5		3		9		Yes^c^		No
	Cancer Australia		72.4		7.7		77.2		4.7		51		Yes^c^		Yes^d^
	NPS^e^ Choosing wisely		76.5		7.5		70.6		6.0		10		Yes		Yes^d^
	Healthdirect		48.0		11.2		80.7		4.2		56		Yes		Yes^d^
	NHS^f^		71.8		7.7		92.3		2.1		20		No		Yes^g,h^
	Ask 3 Questions NHS		85.5		4.8		98.1		2.1		3		Yes^c^		No
	IHI^i^ Ask Me 3		64.6		9.4		100.0		0.5		3		Yes		No
	Ask Share Know		78.8		5.5		75.4		5.2		3		No		No^j^
	Ask 3 Questions Cardiff, UK		78.2		4.8		71.1		5.4		3		Yes^c^		No
	Cancer Council (Australia)		58.7		8.7		77.4		4.6		34		Yes		Yes^h^
	Average of all reputable sites		70.5		7.7		82.8		4.2		19		80% (Yes)		50% (Yes)
**Other sites**															
	Australian Thyroid Foundation		n/a		n/a		70.1		6.3		8		No		No^i^
	American Heart Association (Heart Failure)		56		10.3		82.2		3.8		31		No		No^i^
	HSS^k^ Orthopaedic Hospital		54.7		11.6		64.1		7.5		50		Yes		Yes^d^
	Association for Children’s Mental Health		59.3		9.2		78.3		5.5		30		No		No^j^
	Beyond Blue		50.4		10.8		85.6		3.6		5		No		No^j^
	HealthyWomen.org		73.0		7.7		71.7		5.8		18		Yes		Yes^d,^^h^
	Readers Digest		56.6		10.5		84.9		3.5		12		Yes		Yes^l^
	Psych Central		53.7		10.5		63		7.5		18		Yes^c^		Yes^d,^^h^
	The Foundation for Peripheral Neuropathy		58.2		9.8		64		6.8		23		No		No^j^
	MS^m^ Trust UK		67.2		7.8		85.6		3.5		27		No		Yes^d^
	Average of other websites		56.0		10.3		75.0		5.4		22		40% (Yes)		50% (Yes)
Overall		63.25		9.0		78.9		4.6		20.5		60% (Yes)		50% (Yes)	

^a^FKGL: Flesch-Kincaid Grade Level.

^b^There were no instructions preceding the QPL.

^c^reference.

^d^creation.

^e^NPS: National Prescribing Service.

^f^NHS: National Health Service.

^g^original.

^h^update.

^i^IHI: Institute for Healthcare Improvement.

^j^site copyright date.

^k^HSS: Hospital for Special Surgery.

^l^online publication date.

^m^MS: multiple sclerosis.

## Discussion

### Principal Findings

This environmental scan of the internet for patient QPLs designed for use by patients to ask questions when seeing a doctor identified 173 unique lists on 235 websites. Of the lists found in our search, 15 lists had been used by other websites, with the majority (9/15) of these lists from our original list of reputable websites. The remaining duplicated lists were also from websites we regarded as reputable. In addition, there remained an abundance of advice to users about questions they could, or should, be asking at medical appointments from a wide variety of sources.

We noted that question lists appeared in a wide variety of types of websites, both from the categories of our reputable websites, such as government health information websites (eg, Healthdirect Australia) and disease-specific websites (eg, Cancer Council Australia and America), and from news websites (eg, globalnews.ca), commercial sites and blogs (eg, yourgpsdoc.com), sites of charities (eg, thebraintumourcharity.org), and educational institution websites (eg, sydney.edu.au). URL domain extensions do not always reflect the type of institution publishing the site and materials, yet they influence the user perception of site information [23]. While the source of medical information has not been shown to correlate with the accuracy of medical information [23,34], we noted the majority of lists came from .org extensions, which are generally associated with non-commercial organizations.

While there are detailed and extensive guidelines for the development of decision aids [17], there are currently no accepted standards that apply to QPLs. Many indicators of the quality of patient health information websites have been used in the past, with few variables showing any correlation to information quality, as measured by the accuracy or currency of information presented [34]. Display of creation and update dates, the age of the site, authorship, and URL extension have not been found useful in assessing sites. Our findings of these “quality” indicators were similar to other investigations of online patient materials in these respects. The sample QPL websites had higher rates of display of creation or update dates than those in previous studies of health information on the internet [23,34]. Display of some form of authorship or attribution of the sample materials was also higher than in similar studies of online health resources [23,34]. This is likely due to at least half of the sample being what we regarded as reputable.

In our environmental scan for online QPLs for use when attending a medical appointment, it was unclear in most cases whether doctors endorsed the questions for use in consultations. Prior research has shown that doctor endorsement significantly improves the use of QPLs by patients [4]. There were a couple of exceptions where the lists were placed on the website of particular practitioners where it could be assumed that the doctors in that practice supported patients using the questions included in the lists. In general, however, we were not able to ascertain specific endorsement by medical practitioners for the lists in practice. Further qualitative research to determine doctors’ views on implementing such a wide variety of QPL resources into practice is needed.

Our results show that there is room for improvement in the readability of instructions for using online QPLs. International Patient Decision Aid Standards recommendations for reading grade level for decision aids is a less than an 8th grade level [35]. While all the QPLs in the sample we tested had a grade level that met the International Patient Decision Aid Standards recommendations, accompanying instructional text was often well above this level, especially in our “other” website category.

### Strengths and Limitations

By utilizing the strengths of the environmental scan method, we were able to rapidly assess the online environment, where most Australians look for health information, for the presence of QPLs. While literature reviews have been able to show the benefits of QPLs in practice, no assessment has been made of the number and availability of similar tools in the real-world environment. The online environment changes rapidly, and this scan provides a survey at a point in time of what is available to users. Our search tool was Google.com.au; hence, a similar search strategy in other countries might yield different results. We also limited our search to sites in English.

We noted website updates occurring even as we reviewed the websites; if the scan was to be replicated in the future, the results may be different. Further assessment of the quality of the lists beyond readability would provide additional information. Some proxy measures were used to assess sites, even though the presence of, for example, a date and authorship has been shown to have little correlation with quality. It is not clear which, if any, QPLs we discovered have been evaluated for their efficacy in improving patient and doctor outcomes in consultations.

It is unlikely that Google users might search specifically for question lists using the search terms we used. We were not testing the utility of terms to find QPLs; rather, the intent of our search and choice of search terms were to find as many as possible of the question lists available to users. Research into how users actually access, assess, and use these resources is warranted.

### Conclusions

People seeking information on Google.com.au have a vast number of question lists available to them to use in consultations with their doctor. Surrogate markers of the quality or usefulness of various types of online QPLs have not yet been established, which makes it difficult to assess the value of the abundance of lists. Quality measures for QPLs should follow further assessment of usage and endorsement.

Physician endorsement has been shown to be an important factor in the usefulness of QPLs, but there is little information to suggest whether the QPLs found in this scan would be endorsed by physicians. Whether these diverse QPLs are endorsed by medical practitioners warrants further investigation.

Ensuring that online QPLs and the instructions for their intended use are accessible to patients is important. To improve usage of online QPLs, instructions for their use should have an equivalent readability or at least be accessible to the majority of people in the target groups for which the lists have been created.

## Appendix

Multimedia Appendix 1 Source and URL for QPLs identified.

## Abbreviations

ADHD: attention deficit hyperactivity disorder.
FKGL: Flesch-Kincaid Grade Level.
FRE: Flesch Reading Ease.
HSS: Hospital for Special Surgery.
IHI: Institute for Healthcare Improvement.
MS: multiple sclerosis.
NHS: National Health Service.
NPS: National Prescribing Service.
NSW: New South Wales.
QPL: question prompt list.
SDM: shared decision making.
