# Supplementary material for: Exploring the Vast Choice of Question Prompt Lists Available to Health Consumers via Google: Environmental Scan
Source: J Med Internet Res. 2020 May 29;22(5):e17002. doi: 10.2196/17002 (PMC7293062; doi:10.2196/17002)
Supplement: Multimedia Appendix 1 [file jmir_v22i5e17002_app1.docx]

Supplementary table: Source and URL for QPLs identified

| **Source**  **(Alphabetically)** | **URL** |
| --- | --- |
|  |  |
| 1800 my options | https://www.1800myoptions.org.au/information/getting-abortion-victoria |
| American Association of Retired Persons | https://www.aarp.org/caregiving/health/info-2017/questions-to-ask-doctor.html |
| Advantage care physicians | https://www.acpny.com/patient-info/prepare-for-appointment/questions-for-doctor |
| Agency for Healthcare Research and Quality | https://www.ahrq.gov/professionals/quality-patient-safety/quality-resources/tools/literacy-toolkit/healthlittoolkit2-tool14.html |
| Agency for Healthcare Research and Quality | https://www.ahrq.gov/patients-consumers/patient-involvement/ask-your-doctor/index.html |
| Agency for Healthcare Research and Quality | https://www.ahrq.gov/patients-consumers/patient-involvement/ask-your-doctor/questions-during-appointment.html |
| Alzheimers Assoc | https://www.alz.org/alzheimers-dementia/treatments/questions-for-your-doctor |
| America cancer society - colorectal ca | https://www.cancer.org/cancer/colon-rectal-cancer/detection-diagnosis-staging/talking-with-doctor.html |
| American Academy of Family Physicians | https://familydoctor.org/tips-for-talking-to-your-doctor/ |
| American Academy of Orthopaedic Surgeons | https://orthoinfo.aaos.org/en/treatment/questions-to-ask-your-doctor-before-surgery/ |
| American cancer society | https://cancer.org/treatment/finding-and-paying-for-treatment/choosing-your-treatment-team/questions-to-ask-your-doctor.html |
| American Cancer Society - bladder cancer | https://www.cancer.org/cancer/bladder-cancer/detection-diagnosis-staging/talking-with-doctor.html |
| American cancer society - ovarian ca | https://cancer.org/cancer/ovarian-cancer/detection-diagnosis-staging/talking-with-doctor.html |
| American College of Surgeons | https://www.facs.org/education/patient-education/patient-resources/prepare/10-questions |
| American heart association | http://www.heart.org/en/health-topics/consumer-healthcare/doctor-appointments-questions-to-ask-your-doctor |
| American heart association - general heart health | http://www.heart.org/en/health-topics/cardiac-rehab/communicating-with-professionals/cardiac-rehab-questions-for-your-healthcare-professional |
| American Institute for Cancer Research | http://www.aicr.org/patients-survivors/getting-ready-for-treatment/questions-to-ask-doctor.html |
| American lung association - sarcoid | http://www.lung.org/lung-health-and-diseases/lung-disease-lookup/sarcoidosis/questions-ask-doctor-sarcoidosis.html |
| American Partnership for Eosinophilic disorders | https://apfed.org/resources/for-caregivers/questions-to-ask-your-doctor/ |
| American Psychological Association | http://www.apa.org/helpcenter/questions-doctor.aspx |
| American Psychological Association - PTSD | http://www.apa.org/ptsd-guideline/patients-and-families/questions.aspx |
| American sexual health association | http://www.ashasexualhealth.org/sexual-health/ten-questions-to-ask/ |
| American Society of Clinical Oncology - CancerNet | https://www.cancer.net/navigating-cancer-care/diagnosing-cancer/questions-ask-your-health-care-team |
| Arthritis Foundation | https://www.arthritis.org/Documents/Toolkits/Better-Living-Toolkit/Questions-to-Ask-Your-Doctor.pdf |
| Ask Share Know | http://askshareknow.com.au/ask-questions/ |
| Ask Share Know | https://www.health.org.uk/blog/ask-three-questions |
| Association for Children’s Mental Health | http://www.acmh-mi.org/get-information/childrens-mental-health-101/questions-ask-treatment/ |
| Asthma initiative Michigan pdf - | https://getasthmahelp.org/work-with-doctor.aspx |
| Australian Commission on Quality and Safety in Health Care | https://www.safetyandquality.gov.au/our-work/shared-decision-making/patient-decision-aids/ |
| Australian Red Cross | https://mytransfusion.com.au/blood-transfusion-questions-ask-your-doctor |
| Australian Thyroid Foundation (medication questions) | https://thyroidfoundation.org.au/Questions-to-ask-your-doctor |
| Babble | https://www.babble.com/pregnancy/7-important-questions-to-ask-during-your-first-prenatal-appointment/ |
| Barnes Jewish hospital | https://www.barnesjewish.org/Medical-Services/Heart-Vascular/Heart-Disease-Prevention/Questions-to-Ask-Your-Doctor |
| beyond blue | https://www.beyondblue.org.au/personal-best/pillar/supporting-yourself/questions-to-ask-your-health-professional |
| Beyond five, the face of head and neck cancer - hypopharyngeal ca | https://www.beyondfive.org.au/Types/Hypopharyngeal-cancer/FAQ |
| Bicuspid Foundation .com | http://bicuspidfoundation.com/appointment.htm |
| Bowel Cancer Australia | https://www.bowelcanceraustralia.org/what-i-need-to-ask |
| Breast cancer.org | https://www.breastcancer.org/symptoms/diagnosis/dr_questions |
| Bright focus foundation | https://www.brightfocus.org/macular-glaucoma/news/top-five-questions-ask-your-eye-doctor |
| British heart foundation | https://www.bhf.org.uk/informationsupport/heart-matters-magazine/medical/questions-to-ask-your-doctor |
| Bupa | https://theblueroom.bupa.com.au/healthier/wellness/questions-to-ask-your-health-professional |
| Canada Cancer Society | http://www.cancer.ca/en/cancer-information/cancer-journey/your-healthcare-team/questions-to-ask/treatment-questions/?region=on |
| Canada Patient Safety Institute | http://www.patientsafetyinstitute.ca/en/toolsResources/5-Questions-to-Ask-about-your-Medications/Pages/default.aspx |
| Canadian Cancer Society | http://www.cancer.ca/en/cancer-information/cancer-journey/your-healthcare-team/questions-to-ask/radiation-therapy-questions/?region=on |
| Cancer Australia | https://canceraustralia.gov.au/system/tdf/publications/pcac-cancer-how-are-you-travelling_504af0263f9c9.pdf?file=1&type=node&id=2846 |
| Cancer care | https://www.cancercare.org/publications/243-questions_to_ask_your_health_care_team |
| Cancer center colonoscopy | https://www.cancercenter.com/discussions/blog/seven-questions-you-should-ask-your-doctor-before-getting-a-colonoscopy/ |
| Cancer center lung ca | https://www.cancercenter.com/lung-cancer/questions/ |
| Cancer Council Aust | https://www.cancer.org.au/about-cancer/after-a-diagnosis/questions-to-ask-your-doctor.html |
| Cancer Council Australia | https://www.cancercouncil.com.au/melanoma/diagnosis/health-professionals/ |
| Cancer Council NSW | https://www.cancercouncil.com.au/cancer-information/advanced-cancer/living-with-advanced-cancer/question-checklist/ |
| Cancer Council SA | https://www.cancersa.org.au/information/a-z-index/questions-you-might-like-to-ask-your-doctor |
| Cancer Council SA - lung cancer | https://www.cancersa.org.au/information/a-z-index/questions-to-ask-your-doctor-about-lung-cancer |
| Cancer Council Vic | https://www.cancervic.org.au/cancer-information/treatments/questions_to_ask_your_doctor |
| Cancer of unknown primary Foundation | https://cupfoundjo.org/diagnosis/question-you-might-ask-the-consultant |
| cancer.net | https://www.cancer.net/navigating-cancer-care/teens/questions-ask-your-doctors-and-nurses |
| Cancer.net - link at bottom | https://www.cancer.net/navigating-cancer-care/managing-your-care/questions-ask-when-making-appointments |
| cancer101 | http://cancer101.org/where-do-i-start/questions-to-ask-your-doctor/ |
| Carcinoid/neuro-endo tumour Society Canada | https://cnetscanada.org/patients-caregivers/resources/questions-to-ask-your-doctor/ |
| Cardiosmart - American College of Cardiology | https://www.cardiosmart.org/Heart-Conditions/Heart-Failure/Questions-to-Ask-Your-Doctor |
| Care Search Australia | https://www.caresearch.com.au/caresearch/tabid/3666/Default.aspx |
| Carer Gateway -link to QB | https://www.carergateway.gov.au/talking-to-health-professionals |
| Caresearch , palliative care - links to other sites with q's | https://www.caresearch.com.au/caresearch/tabid/1107/Default.aspx |
| Center for Advancing health | http://www.cfah.org/prepared-patient/communicate-with-your-doctors/asking-your-doctor-questions |
| Choice | https://www.choice.com.au/health-and-body/health-practitioners/doctors/articles/doctor-patient-relationships |
| Choosing Wiesly | http://www.choosingwisely.org.au/resources/consumers/communicating-with-your-healthcare-provider |
| Choosing Wisely | http://www.choosingwisely.org/getting-started/resource-library/additional-materials-for-patients/ |
| Choosing Wisely | http://www.choosingwisely.org.au/resources/consumers/5-questions-to-ask-your-doctor |
| Choosing Wisely | http://www.choosingwisely.co.uk/i-am-a-patient-carer/questions-ask-doctor/ |
| Choosing Wisely | http://www.choosingwisely.org/wp-content/uploads/2018/03/5-Questions-Poster_8.5x11-Eng.pdf |
| Cleveland clinic | https://my.clevelandclinic.org/patients/information/questions-to-ask-your-doctor |
| Consumer reports | https://www.consumerreports.org/doctors/questions-to-ask-your-doctor/ |
| Digital health - federal Communications commission | https://www.fcc.gov/general/five-questions-you-can-ask-your-doctor-about-digital-health |
| Dr Craig Chappell -doctor's site | https://www.in2itmedical.com/blog/9-questions-your-doctor-must-ask-to-treat-low-back-pain |
| Epilepsy action | https://www.epilepsy.org.uk/info/diagnosis/visiting-your-doctor |
| eviQ - link to cancer institute NSW questions | https://www.eviq.org.au/patients-and-carers |
| Family Care Health Inc. | https://www.familycareinc.org/health-news/10-questions-ask-your-doctor/ |
| Family Caregiver Alliance | https://www.caregiver.org/pathways-effective-communication-healthcare-providers-and-caregivers |
| Fighting Blindness charity | http://www.fightingblindness.ie/eye-conditions/healthcare-professionals-who-look-after-your-eyes/questions-to-ask-your-doctor/ |
| Friends of the National Library of Medicine | https://medlineplus.gov/magazine/issues/spring13/articles/spring13pg26-27.html |
| Get Relief Responsibly | https://www.getreliefresponsibly.ca/questions-to-ask-HCP |
| Glaucoma Research Foundation | https://www.glaucoma.org/treatment/working-with-your-doctor.php |
| Global news | https://globalnews.ca/news/3763210/8-questions-you-should-always-ask-your-doctor/ |
| Guardian news referencing JAMA | https://www.theguardian.com/commentisfree/2017/feb/09/three-questions-that-every-patient-should-ask-their-doctor |
| Harbour cancer centre (NZ) | https://www.harbourcancer.co.nz/wp-content/uploads/2017/11/Harbour-Question-List.pdf |
| Harvard Health publishing | https://www.health.harvard.edu/blog/ask-questions-to-get-the-most-out-of-a-health-care-visit-201306146383 |
| Health and Human Services - Vic | https://www.betterhealth.vic.gov.au/health/ServicesAndSupport/Questions-to-ask-your-obstetrician-or-midwife |
| Health Care Consumers -links to CW5, AM3 and own questions | https://www.hcca.org.au/consumers/questions-to-ask-your-health-professional/ |
| Health Care Consumers Queensland | http://www.hcq.org.au/for-health-consumers-carers/being-involved-in-your-care/ |
| Health Consumers Alliance SA | https://www.hcasa.asn.au/health-literacy/health-literacy-resources-for-consumers |
| Health in Aging - dementia | http://www.healthinaging.org/resources/resource:dementia-what-to-ask/ |
| Health Service Executive | https://www.hse.ie/eng/services/yourhealthservice/focus/ask.html |
| Health translation publication | http://www.healthtranslations.vic.gov.au/bhcv2/bhcht.nsf/PresentDetail?Open&s=Question_prompt_lists_for_people_with_cancer_-_Haematologist_Question_List |
| HealthDay health news site | https://consumer.healthday.com/encyclopedia/diabetes-13/misc-diabetes-news-181/questions-to-ask-your-doctor-diabetes-644932.html |
| Healthdirect | https://www.healthdirect.gov.au/questions-to-ask-your-doctor |
| Healthdirect | https://www.healthdirect.gov.au/how-to-find-the-right-health-professional |
| Healthdirect - surgical | https://www.healthdirect.gov.au/questions-to-ask-before-surgery |
| Healthline - menopause | https://www.healthline.com/health/menopause/talking-ob-gyn |
| Healthline - parent of child with CF | https://www.healthline.com/health/cystic-fibrosis/ddg-questions-child-cf |
| Healthline - psoriasis | https://www.healthline.com/health/psoriasis/moderate-to-severe/questions-your-dermatologist-wants-you-to-ask-about-psoriasis |
| Healthline.com | https://www.healthline.com/health/copd/questions-doctor |
| Healthlink British Colombia | https://www.healthlinkbc.ca/health-topics/hw226888 |
| Healthy Women -women mid life | https://www.healthywomen.org/content/article/health-your-50s-questions-ask-your-health-care-professional |
| Heart foundation | http://www.heart.org/en/health-topics/cardiac-rehab/communicating-with-professionals/preparing-for-medical-visits |
| Heart Foundation Australia | https://www.heartfoundation.org.au/after-my-heart-attack/heart-attack-treatment |
| Henry Ford health system - cancer | http://www.henryfordlivewell.com/5-questions-cancer-patients-ask-doctor/ |
| Hepatitis Australia | https://www.hepatitisaustralia.com/questions-to-ask-your-doctor/ |
| Hepatitis Australia Inc | http://testcurelive.com.au/questions/ |
| Her Money from Jean Chatzsky | https://www.dailyworth.com/posts/10-questions-ask-doctor-major-surgery |
| Home support services | https://www.hss.com.au/blog/hss-promotes-choosing-wisely-australia-5-questions-to-ask-resource/ |
| HSS - orthopedic hospital | https://www.hss.edu/conditions_questions-to-ask-doctor.asp |
| Institute for healthcare improvement | http://www.ihi.org/resources/Pages/Tools/Ask-Me-3-Good-Questions-for-Your-Good-Health.aspx |
| Interior Health. Canada | https://www.interiorhealth.ca/YourCare/MentalHealthSubstanceUse/MentalHealth/Documents/SurvivalKit_AskingQuestions.pdf |
| Joe Niekro Foundation charity Surgery FU neuro (AVMs etc) | https://www.joeniekrofoundation.com/aneurysms/questions-to-ask-your-doctor-after-surgery/ |
| John Hopkins - surgery | https://www.hopkinsmedicine.org/healthlibrary/conditions/surgical_care/questions_to_ask_before_surgery_85,P01409 |
| John Torinus - doctor's site | http://johntorinus.com/ten-questions-to-ask-your-doctor/ |
| Journey care - private hosp/clinics | https://journeycare.org/questions-ask-doctor/ |
| Kidney Cancer Canada | https://www.kidneycancercanada.ca/for-patients-and-caregivers/questions-to-ask-your-doctor/ |
| Kids Health .org | https://kidshealth.org/en/teens/questions-doctor.html |
| Leukaemia & lymphoma society | https://www.lls.org/managing-your-cancer/communicating-with-your-specialist/printable-question-guides |
| Leukaemia foundation | https://www.leukaemia.org.au/disease-information/living-with-myeloma/questions-to-ask-your-doctor/ |
| LGBTIQ Healthcare Guild | http://healthcareguild.com/questions_to_ask_providers.html |
| Lung cancer .org | https://www.lungcancer.org/find_information/publications/163-lung_cancer_101/277-questions_to_ask |
| Lungevity | https://lungevity.org/for-patients-caregivers/asking-right-questions/questions-to-ask-your-healthcare-professionals |
| Lungevity | https://lungevity.org/for-patients-caregivers/asking-right-questions |
| Lupus rebel | https://lupusrebel.com/what-questions-should-you-ask-on-your-visit-to-the-doctor/ |
| MacMillan cancer support | https://www.macmillan.org.uk/information-and-support/treating/treatment-decisions/questions-to-ask |
| Macular Disease Foundation Australia | https://www.mdfoundation.com.au/content/talking-your-eye-health-professional-0 |
| Marie Curie | https://www.mariecurie.org.uk/help/support/diagnosed/recent-diagnosis/doctors-appointments |
| Mater hospital | https://www.mns.org.au/patients-visitors/being-involved-in-your-care/questions-to-ask-your-doctor |
| Mather Vision Group | https://mathervisiongroup.com/questions-to-ask-an-eye-doctor/ |
| Medala foundation | http://www.medelabreastfeedingtips.ca/6-questions-to-ask-your-healthcare-professional-to-avoid-common-breastfeeding-problems/ |
| Medibank | https://www.medibank.com.au/livebetter/my-medibank/healthcare/asking-the-right-questions/ |
| Medical psychology research unit The University of Sydney | http://www.psych.usyd.edu.au/cemped/docs/comms_Booklet1_printable.pdf |
| MedicineNet.com | https://www.medicinenet.com/questions_to_ask_your_doctor_-_general/views.htm |
| Medisafe - medications | https://medisafe.com/5-questions-every-patient-should-ask-their-doctor-when-prescribed-a-med/ |
| Medline Plus - immunotherapy | https://medlineplus.gov/ency/patientinstructions/000828.htm |
| Medline Plus - T2DM | https://medlineplus.gov/ency/patientinstructions/000217.htm |
| MensHealth.com | https://www.menshealth.com/health/a19537757/10-questions-you-must-ask-your-doctor/ |
| Mental Health America | http://www.mentalhealthamerica.net/questions-ask-provider |
| Metastatic breast cancer network | http://www.mbcn.org/questions-to-ask-your-doctor/ |
| Million hearts - cvd | https://millionhearts.hhs.gov/files/his_tips_for_communicating_hcp.pdf |
| Mommy bites | https://mommybites.com/col1/baby/questions-to-ask-at-first-newborn-doctor-visit/ |
| Monash University | https://www.monash.edu/medicine/sphpm/mchri/pcos/resources/pcos-question-prompt-list |
| Monash University | https://www.monash.edu/__data/assets/pdf_file/0009/1401768/PCOS-QPL.pdf |
| Motherly - antenatal | https://www.mother.ly/life/9-essential-questions-to-ask-at-your-first-prenatal-visit |
| MS Trust UK | https://www.mstrust.org.uk/a-z/questions-ask-health-professionals |
| Multiple sclerosis trust | https://www.mstrust.org.uk/about-ms/ms-treatments/making-most-appointments |
| National Anstitute on Aging | https://www.nia.nih.gov/health/what-should-i-ask-my-doctor-during-checkup |
| National Cancer Institute | https://www.cancer.gov/about-cancer/diagnosis-staging/questions |
| National CML Society | http://www.nationalcmlsociety.org/questions-ask-your-doctor |
| National Jewish Health | https://www.nationaljewish.org/patients-visitors/patient-info/prepare-for-your-appointment/doctor-may-ask |
| National Prescribing Service - choosing wisely | http://www.choosingwisely.org.au/getmedia/22343835-8b00-454c-a540-4d5f622efa19/5-questions-to-ask-your-doctor-before-you-get-any-test-treatment-or-procedure.pdf.aspx |
| Nebraska Coalition for Patient Safety | https://www.nepatientsafety.org/ask-questions |
| Needy Meds | http://www.bemedwise.org/medical-questions/why-health-questions-matter |
| Needy Meds - paracetamol | http://www.bemedwise.org/acetaminophen/questions-healthcare-professional |
| New York State Department of Health - palliative care | https://www.health.ny.gov/professionals/patients/patient_rights/palliative_care/2012-06-26_proposed_questions_answers.htm |
| NHS | https://www.nhs.uk/using-the-nhs/nhs-services/gps/what-to-ask-your-doctor/ |
| NHS | http://www.pat.nhs.uk/downloads/patient-information-leaflets/other/Ask%203%20Questions%20Leaflet.pdf |
| Novant Health - AM3 | https://www.novanthealth.org/home/quality--safety/ask-me-3.aspx |
| NPR.org (media) | https://www.npr.org/sections/health-shots/2011/09/20/140643614/ten-questions-to-ask-you-doctor |
| NSW health | https://www.health.nsw.gov.au/Hospitals/Going_To_hospital/Pages/questions.aspx |
| One needle, One syringe, only one time | http://www.oneandonlycampaign.org/content/what-ask-healthcare-providers |
| Own your health - palliative care | http://www.ownyourhealthwa.org/making-decisions-about-treatment/5-questions-to-ask-when-your-doctor-suggests-a-treatment/ |
| Palliative care Australia | http://palliativecare.org.au/resources/questions-for-carers-to-ask-the-team-caring-for-your-loved-one |
| Palliative Care Australia | http://palliativecare.org.au/asking-questions |
| Palliative Care Australia | http://palliativecare.org.au/resources/asking-questions |
| Palliative Care Australia | http://palliativecare.org.au/wp-content/uploads/2015/05/PCA002_Asking-Questions-Can-Help_FA.pdf |
| Patient Safety Action Network | https://safepatientproject.org/posts/5366-6-questions-to-ask-before-getting-a-ct-scan-or-x-ray |
| PBS Australia | https://www.pbs.org/newshour/economy/column-6-questions-to-ask-at-every-doctors-appointment |
| Pharmacy - IVF | https://www.kingsrx.com/what-are-good-questions-to-ask-during-an-ivf-consult/ |
| Pregnancy Birth and Baby | https://www.pregnancybirthbaby.org.au/questions-to-ask-your-doctor-about-tests-and-scans |
| Primary Healthcare Network Northern Sydney - GP | https://sydneynorthhealthnetwork.org.au/communityhealth/questions-to-ask-your-gp/ |
| Private cosmetic dermatologist | https://www.allure.com/story/exact-questions-to-ask-cosmetic-dermatologists |
| Private dermatology | https://www.epiphanydermatology.com/blog/5-questions-patient-should-ask-dermatologist/ |
| Providence Health Plan, LBP, insurance site | https://healthplans.providence.org/fittogether/find-your-fit/manage-health-conditions/low-back-pain/low-back-pain-questions-and-symptoms/ |
| Psoriasis Speaks (AbbVie) | https://www.psoriasis.com/questions-to-ask-your-doctor |
| Psych central | https://psychcentral.com/lib/questions-for-your-doctor-about-medications/ |
| Pulmonary associates - lung | https://floridachest.com/pulmonary-blog/9-questions-to-ask-your-pulmonologist-before-your-first-appointment |
| Quality Improvement Organizations | https://qioprogram.org/ask-your-doctor-questions |
| Question builder - AHRQ | https://www.ahrq.gov/patients-consumers/question-builder.html |
| Reader's digest - diabetes | https://www.rd.com/health/conditions/diabetes-questions/ |
| Readers digest | https://www.rd.com/health/wellness/ask-your-dermatologist-next-checkup/ |
| Readers Digest - general | http://time.com/4433153/9-questions-ask-doctor/ |
| Renal Support Network | https://www.rsnhope.org/health-library/top-8-questions-to-ask-your-nephrologist/ |
| FDA radiology | https://www.fda.gov/radiation-emittingproducts/radiationemittingproductsandprocedures/medicalimaging/medicalx-rays/default.htm |
| Richard and Annette Bloch Foundation - cancer charity | http://blochcancer.org/2009/12-questions-for-a-newly-diagnosed-cancer-patient-to-ask-their-physician/ |
| RT Answers - radiotherapy | http://www.rtanswers.org/Questions-to-Ask-Your-Doctor/ |
| Scarborough physio and health | https://physioredcliffe.com.au/4-things-you-must-ask-your-health-professional%E2%80%8F/ |
| Scottish Health Council | http://www.scottishhealthcouncil.org/patient__public_participation/participation_toolkit/ask_me_3.aspx |
| Script your future | http://www.scriptyourfuture.org/questions-to-ask-your-health-care-professional/ |
| Seconds count - The Society for Cardiovascular Angiography and Interventions | http://www.secondscount.org/heart-resources/heart-resources-detail-2/questions-to-ask-your-doctor-about-stroke |
| Sentara (hosptials) | https://www.sentara.com/patientguide/becoming-a-patient/questions-to-ask-your-doctor.aspx |
| Sing health - cancer | https://www.singhealth.com.sg/Health-Information/Pages/Ask-Doctor.aspx |
| Society of cardiothoracic surgeons | https://ctsurgerypatients.org/five-questions-i-wish-my-patients-would-ask-me |
| St Vincent’s Brisbane private hospital | https://www.svphb.org.au/patients-visitors/being-involved-in-your-care/questions-to-ask-your-doctor |
| St Vincent’s hospital | https://www.svha.org.au/our-care/being-involved-in-your-care/questions-to-ask-your-doctor-and-health-care-team |
| Susan G Komen | https://knowyourgirls.org/resources/questions-to-ask-your-doctor-about-breast-health/ |
| Susan G Komen breast cancer foundation | http://ww5.komen.org/BreastCancer/QuestionstoAsktheDoctorPDFDownloads.html |
| Sydney Morning Herald | https://www.smh.com.au/opinion/the-question-every-patient-should-ask-their-doctor-20160113-gm4mgt.html |
| Sydney Morning Herald | https://www.smh.com.au/healthcare/the-five-questions-you-should-always-ask-your-doctor-20161215-gtbnx7.html |
| The Blue Room - Bupa, heart failure | https://theblueroom.bupa.com.au/manage-and-recover/heart-health/questions-to-ask-about-heart-failure |
| The Brain Tumour Charity | https://www.thebraintumourcharity.org/understanding-brain-tumours/navigating-the-system/questions-to-ask/ |
| The Bump | https://www.thebump.com/a/what-to-ask-my-ob-at-my-first-appointment |
| The Foundation for Peripheral Neuropathy | https://www.foundationforpn.org/what-is-peripheral-neuropathy/patient-education/ |
| The Health Literacy Place - AM3 link | http://www.healthliteracyplace.org.uk/tools-and-techniques/encouraging-patient-questions/ |
| The University of Sydney (Psychology) | http://www.psych.usyd.edu.au/cemped/com_question_prompt.shtml |
| The University of Sydney (Psychology) | https://sydney.edu.au/science/our-research/research-centres/centre-for-medical-psychology.html |
| The University of Sydney news | https://sydney.edu.au/news-opinion/news/2016/01/15/the-question-every-patient-should-ask-their-doctor.html |
| The University of Texas MD Anderson Cancer Center | https://www.mdanderson.org/publications/cancerwise/2013/06/newly-diagnosed-cancer-patients-questions-to-ask-your-health-car.html |
| Think mental health WA | https://www.thinkmentalhealthwa.com.au/supporting-my-mental-health/reasons-to-seek-help/questions-to-ask-your-gp/ |
| Think Mental Health WA | https://www.thinkmentalhealthwa.com.au/mental-health-support-services/how-your-gp-can-help/questions-to-ask-your-gp/ |
| UC San Diego Health | https://health.ucsd.edu/news/features/Pages/2014-10-31-5-questions-to-ask-your-doc.aspx |
| UCLA brain tumor centre | https://www.uclahealth.org/braintumor/questions-to-ask-your-doctor |
| University of California San Francisco | https://www.ucsfhealth.org/education/womens_health_checklist_questions_to_ask_your_provider/ |
| University of California San Francisco - cancer questions | https://www.ucsfhealth.org/education/questions_to_ask_your_doctor/ |
| University of Maryland | https://www.umms.org/shore/patients-visitors/for-patients/patient-safety-quality/resources/questions-ask-your-doctor |
| University of Memphis - cancer | http://www.memphis.edu/chlhc/resources/questionpromptlistforpatientsandcaregivers.php |
| University of Pittsburgh Medical Centre | http://www.upmc.com/patients-visitors/education/older-adults/Pages/questions-all-patients-should-ask-pharmacist.aspx |
| UPMC | https://share.upmc.com/2017/07/questions-to-ask-your-doctor/ |
| US Department of VA - HIV | https://www.hiv.va.gov/patient/treat/HIVdrugs-questions-to-ask.asp |
| US News | https://health.usnews.com/health-news/patient-advice/slideshows/10-questions-doctors-wish-their-patients-would-ask |
| US veterans affairs re HIV/veterans | https://www.hiv.va.gov/patient/diagnosis/questions-for-doctor.asp |
| Vision aware | http://www.visionaware.org/info/your-eye-condition/eye-health/questions-you-should-ask/125 |
| WebMD | https://www.webmd.com/cholesterol-management/guide/questions-doctor-cholesterol |
| WebMD - arthritis | https://www.emedicinehealth.com/arthritis_questions_to_ask_your_doctor/article_em.htm |
| WebMD - lupus | https://www.webmd.com/lupus/questions-doctor-lupus |
| WebMD - schizophrenia | https://www.webmd.com/schizophrenia/10-questions-to-ask-your-doctor-about-schizophrenia |
| WebMed - depression | https://www.webmd.com/depression/12-questions-ask-doctor |
| Wikipedia links to choosing wisely | https://en.wikipedia.org/wiki/Doctor%E2%80%93patient_relationship |
| Wiser healthcare | https://www.wiserhealthcare.org.au/questions-to-ask-a-health-professional/ |
| Young minds UK | https://youngminds.org.uk/find-help/your-guide-to-support/questions-you-should-ask/ |
| Your GPS Doc - blog | https://yourgpsdoc.com/2017/10/02/3-questions-must-ask-doctor-part-1/ |
| Your mental health.ie | http://www.yourmentalhealth.ie/mind-yourself/concerned/your-mental-health/questions/ |
